# Epidermal inclusion cyst in male breast: how to differentiate from other male breast lesions

**DOI:** 10.1016/j.radcr.2022.07.097

**Published:** 2022-08-12

**Authors:** Murat Ak, Cagri Yurtsever, Omer F. Cakir, Nalan Yurtsever

**Affiliations:** a Department of Radiology, University of Pittsburgh Medical Center, 5115 Centre Ave, Pittsburgh, PA 15232, USA; b Department of Radiology, NYC Health Hospitals, Harlem, NY, USA; c Department of Radiology, Sultan Abdulhamid Han Teaching Hospital, Istanbul, Turkey; d Department of Anatomic and Clinical Pathology, Hofstra Northwell School of Medicine, NY, USA

**Keywords:** Epidermal inclusion cyst, Male breast, Gynecomastia

## Abstract

Male breast lesions are relatively less common. The most encountered malignant lesion in the male breast is ductal adenocarcinoma; and benign lesions are gynecomastia, fibrocystic disease, intramammary lymph node, fibroadenoma, lipoma and epidermal inclusion cyst (EIC), respectively [5,6]. To date, there had been published only a few cases of EIC of the male breast in literature [3,5,6]. In this case, we aimed to present a new case of EIC with its clinical, radiological and pathological characteristics in the male breast. It had benign sonographic and magnetic resonance imaging findings but had also malignant imaging findings with diffusion restriction on diffusion-weighted imaging.

## Case report

A 51-year-old male presented with a slowly growing lesion posterior to the left nipple for 3 years. The patient noted pain with palpation but denied fevers, chills, fatigue, weight loss, and nipple discharge. The patient denied any trauma, infection, bug bite, skin condition, new medication use or past surgical procedure. The patient had no significant medical history other than DM and CKD with EGFR < 30 mL/min/1.73 m^2^. He had no known family history of breast or ovarian cancer. On physical examination, a mobile, painless, approximately 3 cm mass was palpated in the posterior of the left nipple with no associated skin changes. Shrinkage or other skin changes that may be considered as a sign for malignancy were observed neither on the skin nor on the nipple. Physical examination of axilla was normal.

**Fig. 1:**
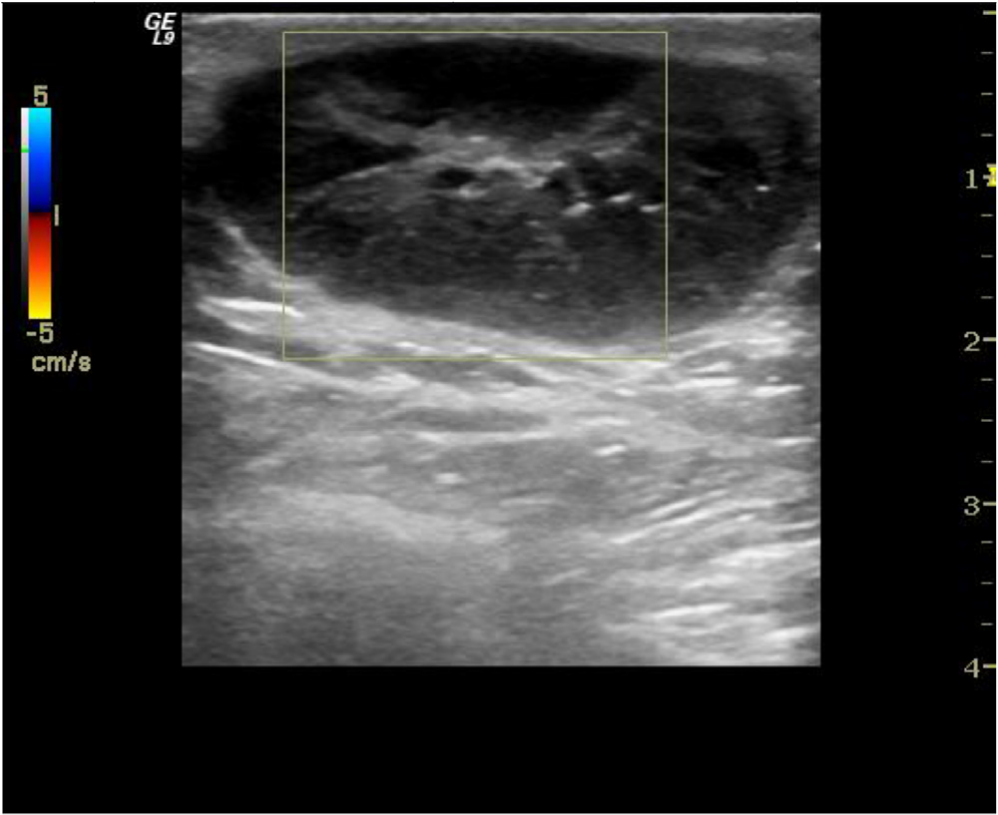
Axial sonogram shows well-circumscribed, hypoechoic lesion with posterior acoustic enhancement in the posterior of nipple. No vascular signal was noted in color box.

Mammogram was declined and initial ultrasonography revealed a superficial, well-circumscribed, ovoid-shaped, and hypoechoic lesion in the posterior nipple. It was parallel to the skin, and the long dimension was 34 mm (Bi-Rads 4a). Posterior acoustic enhancement was detected on grayscale imaging. There was no vascular sign-on Doppler imaging (Fig. 1). The axilla was free of lymphadenopathy.

**Fig. 2:**
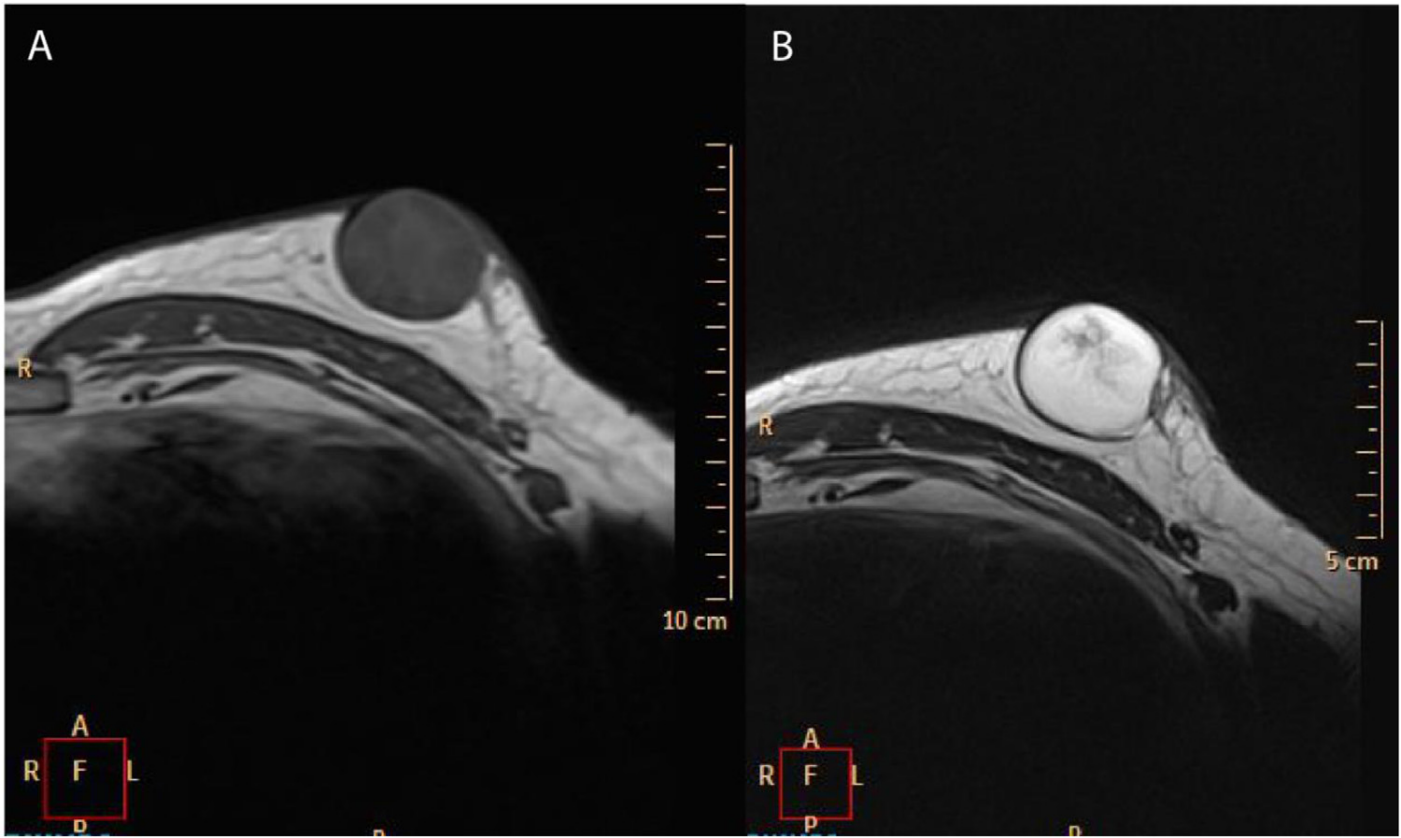
On magnetic resonance imaging (MRI); (a) lesion appeared mild hypointense on T1-weighted imaging, (b) hyperintense on T2-weighted imaging.

**Fig. 3:**
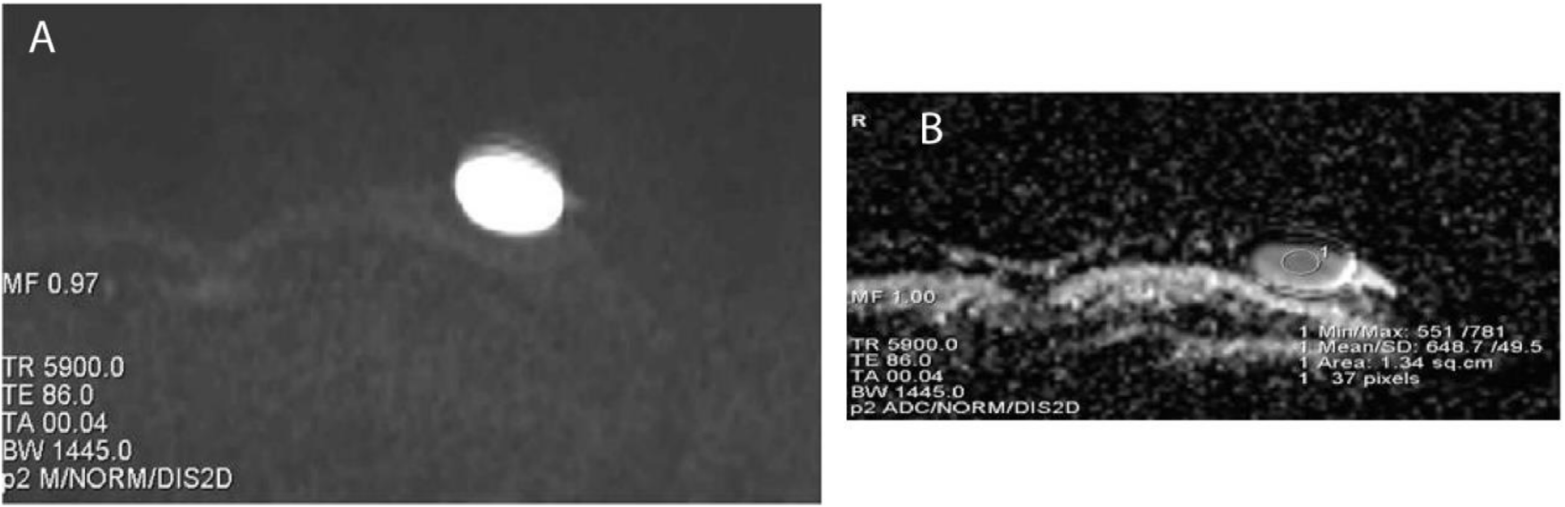
On diffusion weighted imaging (DWI), lesion showed markedly diffusion restriction with ADC value of 0.7 × 10^−3^ s/mm^2^ (A: DWI, B: ADC Map).

**Fig. 4:**
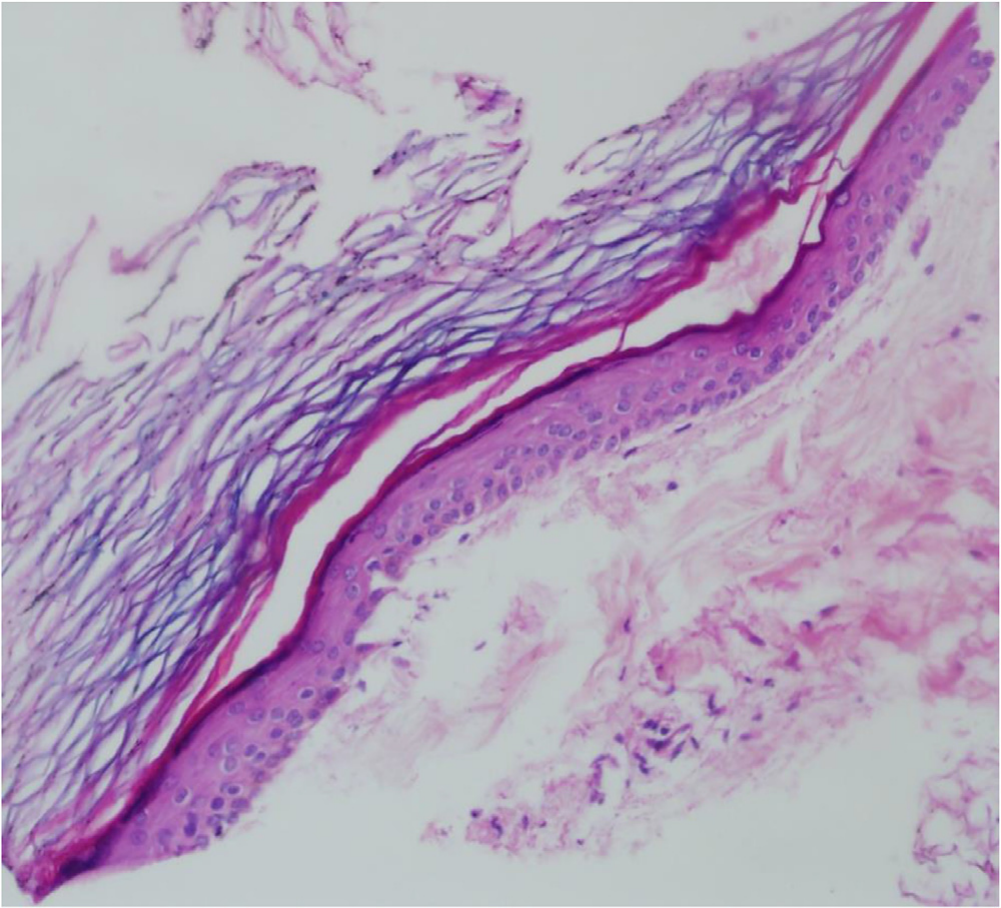
The photomicrograph shows that the lining is composed of a bland appearing squamous epithelium and the contents of the epidermoid cyst consist of laminated ortho-keratotic material.

On magnetic resonance imaging (MRI), the lesion appeared mild hypointense on T1-weighted imaging and hyperintense on T2-weighted imaging (Figs. 2a and b). On diffusion-weighted imaging (DWI), the lesion showed markedly diffusion restriction with ADC value of 0.7 × 10^−3^ s/mm^2^ (Figs. 3a and b). US-guided core needle biopsy was performed. Pathological examination of the biopsy specimen demonstrated fibroadipose tissue with a cyst with no obvious masses suggesting a benign cystic lesion (Fig. 4). Then, the lesion was surgically removed, and the core biopsy histologically confirmed.

## Discussion

EIC is a benign cutaneous lesion that represents the proliferation and implantation of squamous epithelium within a circumscribed space in the dermis or sub-dermis [1,2]. Typical locations for EIC are face, neck, trunk region, and the extremities [1,2]. In the etiology of EIC, there is obstruction or occlusion of hair follicles and skin trauma due to insect bite or surgical intervention [1,2]. Potential complications of EIC are spontaneous rupture and abscesses [3]. EIC is considered as a rare cause of breast lesion in females and even rarer in the male breast. It is assumed that EIC in the breast is more prevalent than observed, but medical attention is not usually focused on this issue due to small and painless swelling [3].

Clinically, it causes a painless, fixed or mobile lump or swelling in the breast. On ultrasonography, a hypoechoic, well-circumscribed lesion which is close to the epidermis, is observed. Because of its cystic nature, posterior acoustic enhancement is notable without any internal vascularization on Doppler imaging.

The differential diagnosis of male breast lump includes benign lesions such as gynecomastia, fibrocystic change, fibroadenoma, hematoma, abscess, and lipoma and malignant lesions such as ductal carcinoma, metastasis, and lymphoma [4,5].

Gynecomastia is the most common benign lesion of the male breast. Diffuse and nodular gynecomastia show slow initial and persistent enhancement with normal-appearing parenchymal architecture on MRI. Gynecomastia looks like an irregularly contoured hypoechoic lesion or disk-shaped hypoechoic nodule surrounded by the fatty tissue around in the posterior of the areola. On the contrary, EIC has a well-circumscribed contour and notable posterior acoustic enhancement. The sonographic image of fibrocystic change is gynecomastia-based dilatation of ductal structures [4]. Cyst in fibrocystic changes usually appears anechoic. Fibroadenoma usually develops in a gynecomastia base, and on sonography, it appears to be a well-circumscribed hypoechoic lesion without posterior acoustic enhancement [4]. Fibroadenoma shows benign morphologic characteristics and non-enhancing septations on MRI and is presumed to have a higher ADC value on DWI [6,7]. Intramammary lymph nodes (IMLN) are usually located in the upper outer quadrant of the breast. On mammography, oval or reniform with denser peripheral margins represent the cortex and more lucent centers representing fat in the hilum. The sonographic feature of the IMLN is an oval or reniform mass with a hypoechoic cortex and a hyperechoic hilum [8]. Lipoma appears to be a well-circumscribed, encapsulated, and hyperechogenic lesion on sonography. Also, MRI is the most sensitive imaging modality in diagnosing lipoma with hyperintense on T1- and T2-weighted imaging and signal loss with fat suppression.

Diffusion-weighted images measure the mobility of water molecules within the tissue, reflecting the cellular microenvironment [9]. Hematoma, breast abscess, may show diffusion restriction [10]. Hemorrhage shows a high signal on DWI and reduced ADC values. Our patient did not have any trauma history and had this lump for a long time. Breast abscess also shows a high signal on DWI and reduced ADC values [11]. But generally shows an ill-defined heterogeneous collection associated with hyperemia around the abscess on Doppler US and skin thickening on the US [11]. But our patient did not have any signs of infection, skin thickening, or perilesional hyperemia.

Male breast cancer accounts for approximately % 1 of all male breast lesions. The sonographic feature of male breast cancer is a non-parallel, hypoechoic lesion with an irregular contour, similar to female breast malignancy [8]. Unlike lesions of the female breast, well-circumscribed lesions must be evaluated carefully in men, due to a higher probability of malignancy [4,8].

On DWI, breast cancers often have decreased diffusivity and appear hyperintense compared to adjacent tissues [9]. They generally show ill-defined contours and internal Doppler signals on US [12]. Our patient had a well-defined lesion with no internal Doppler signals on US. Intraductal papilloma is an uncommon benign tumor that can affect males of any age. It presents clinically as a palpable lump that is typically unilateral, painful or not, and may or may not have papillary discharge [13]. On Doppler US, the primary sonographic findings are an intraluminal mass with dilated duct and inner hypervascularization [13]. Intraductal papilloma shows a high signal on DWI and reduced ADC values [13]. Our patient's lesion was not related to ducts and did not show inner vascularization on Doppler US (Table 1).

**Table 1:** Radiological features of male breast lesions.

Lesions in Male Breast	Sonographic features	Mammographic features	MRI features
Ductal carcinoma	Eccentric location, irregular shaped hypoechoic solid mass, spiculated contours, doppler demonstrate internal vessels	Irregular shaped, spiculated/lobulated margins, high density	T1 C (+); irregular enhancement DWI; diffusion restriction
Gynecomastia	Subareolar location, disc/irregular shaped hypoechoic area	Variable*	Variable*
Fibroadenoma	Well-circumscribed, round to ovoid, hypoechoic mass (associated with or accompanied by gynecomastia)	Hypodense/isodense, may contain calcification	T1; hypointense/isointense T2; hypointense/hyperintense T1 (C+); nonenhancing internal septations, slow internal enhancement
Lipoma	Well-circumscribed mass, hypo/iso/hyperechoic with thin hyperechoic capsule	Radiolucent, fat density	T1, T2; hyperintense, signal loss with fat suppression
Epidermal inclusion cyst	Well-circumscribed subareolar hypoechoic mass, close location to epidermis, no vascular signal on doppler	High density adjacent to skin	T1; mild hypointense T2; hyperintense DWI; diffusion restriction
Intramammary lymph node	Oval shaped, reniform, hypoechoic cortex- hyperechoic hilum	Denser peripheral margin(cortex), more lucent center (hilum)	Not a specific feature
Breast abscess	Hypoechoic collection, mostly multiloculated, no vascularity within the collection, posterior acoustic enhancement due to fluid content an echogenic, vascular rim	Skin thickening and asymmetric density, mass or distortion	High signal on DWI and reduced ADC

In the treatment of EIC, surgical excision is advised in order to avoid possible complications such as cyst rupture, abscess, and potential of malignant transformation [1,^3^,^14^,15].

As a conclusion, EIC is a rare benign breast lesion in males. DWI is useful in terms of differentiating epidermal inclusion cyst from other breast lesions in addition to other MRI features.

## Patient consent

I declare the informed consent for publication has been obtained.
